# Supplementary material for: RodZ modulates geometric localization of the bacterial actin MreB to regulate cell shape
Source: Nat Commun. 2018 Mar 29;9:1280. doi: 10.1038/s41467-018-03633-x (PMC5876373; doi:10.1038/s41467-018-03633-x)
Supplement: Supplementary file 1 — Supplementary Information(PDF 1188 kb) [file 41467_2018_3633_MOESM1_ESM.pdf]

Supplementary Information for "RodZ modulates geometric localization of the bacterial actin MreB to regulate cell shape"

Alexandre Colavin[1,*], Handuo Shi[2,*], Kerwyn Casey Huang[1,2,3,4,†]

[1]Biophysics Program, Stanford University, Stanford, CA 94305, USA

[2]Department of Bioengineering, Stanford University, Stanford, CA 94305, USA

[3]Department of Microbiology and Immunology, Stanford University, Stanford, CA 94305, USA

[4]Corresponding author

[*]These authors contributed equally.

[†]Corresponding author: Kerwyn Casey Huang (kchuang@stanford.edu)

Supplementary Tables

Supplementary Table 1: Description of strains used in this study. MG1655 is background genotype for all strains.

Strain	Genotype	Source
MG1655	N/A	Laboratory stock
NO34	*csrD::kan*, *mreB::msfGFP-mreB*	[1]
KC356	P*mreB'::msfGFP-mreB'-CD* on pSC101 plasmid (pRM102).	This work
KC507	*csrD::kan*, *mreB'::msfGFP-mreB'-E276D*	[1]
KC708	NO34 with *rodZ::kan* by P1 transduction from KEIO library strain JW2500	This work and ref.[2]
KC707	KC507 with *rodZ::kan* by P1 transduction from KEIO library strain JW2500	This work and ref.[2]
KC694	KC708 + plasmid P*mreB'::msfGFP-mreB'-E276D-CD*	This work
KC717	NO34 + P*rodZ*<>(*frt araC* P_{BAD})	This work and ref.[3]
KC692	KC708 + plasmid P*mreB'::msfGFP-mreB'-CD*	This work
KC710	KC707 + plasmid P*mreB'::msfGFP-mreB'-E276D-CD*	This work
KC967	*csrD::kan, mreB'::msfGFP-mreB'-D83E*	This work
KC968	*csrD::kan, mreB'::msfGFP-mreB'-R124C*	This work
KC969	*csrD::kan, mreB'::msfGFP-mreB'-A174T*	This work

Supplementary Table 2: Strain length and width.

Strain Name	Genotype	Condition	Figure	Mean length (μm)	Mean width (μm)
KC356	P*mreB'::msfGFP-mreB'-CD* on pSC101 plasmid (pRM102).	Stationary phase (LB, 24 h)	1e,f,h	1.54	1.05
		15 min outgrowth in fresh LB	1h	1.51	1.03
		30 min outgrowth in fresh LB	1h	1.64	1.10
		45 min outgrowth in fresh LB	1h	1.94	1.16
		60 min outgrowth in fresh LB	1h	2.47	1.57
		75 min outgrowth in fresh LB	1h	2.93	1.46
		90 min outgrowth in fresh LB	1e-h, 3, and 5b	3.48	1.13
		105 min outgrowth in fresh LB	1h	3.55	1.10
		120 min outgrowth in fresh LB	1h	3.57	1.10
		135 min outgrowth in fresh LB	1h	3.51	1.08
		150 min outgrowth in fresh LB	1h	3.12	1.04
		165 min outgrowth in fresh LB	1h	2.61	1.01
		180 min outgrowth in fresh LB	1h	2.33	0.99
		195 min outgrowth in fresh LB	1h	2.33	0.97
KC717	*csrD::kan*, *mreB::msfGFP-mreB* + P*rodZ*<>(*frt araC* P_{BAD})	Stationary phase (LB, 12h, 0.2% Ara)	2b	4.54	1.69
		Stationary phase (LB, 12h, 0% Ara)	2b	4.35	3.83
		Stat. phase (0% Ara) + 60 min incubation (0% Ara)	2d	3.30	1.94
		Stat. phase (0% Ara) + 60 min incubation (0.01% Ara)	2d	3.84	1.76
		Stat. phase (0% Ara) + 60 min incubation	2d	3.29	1.76

		(0.02% Ara)			
		Stat. phase (0% Ara) + 60 min incubation (0.05% Ara)	2d	3.61	1.77
		Stat. phase (0% Ara) + 60 min incubation (0.10% Ara)	2d	3.86	1.75
		Stat. phase (0% Ara) + 60 min incubation (0.20% Ara)	2d	3.61	1.77
		Stat. phase (0% Ara) + 60 min incubation (1% Xyl)	2d	4.20	1.93
		4 h outgrowth in fresh LB 1:10,000 dilution (0% Ara)	2e	3.57	1.94
		4 h outgrowth in fresh LB 1:10,000 dilution (0.005% Ara)	2e	4.41	1.72
		4 h outgrowth in fresh LB 1:10,000 dilution (0.01% Ara)	2e	3.92	1.44
		4 h outgrowth in fresh LB 1:10,000 dilution (0.02% Ara)	2e	3.41	1.41
		4 h outgrowth in fresh LB 1:10,000 dilution (0.05% Ara)	2e	4.07	1.42
		4 h outgrowth in fresh LB 1:10,000 dilution (0.1% Ara)	2e	3.99	1.44
		4 h outgrowth in fresh LB 1:10,000 dilution (0.2% Ara)	2e	4.74	1.47
KC507	*csrD::kan*, *mreB'::msfGFP-mreB'-E276D*	90 min outgrowth in fresh LB	3, 5f	3.71	1.17
NO34	*csrD::kan*, *mreB::msfGFP-mreB*	90 min outgrowth in fresh LB	3, 5f	3.77	1.21
KC967	*csrD::kan, mreB'::msfGFP-mreB'-D83E*	105 min outgrowth in fresh LB	3, 5f	3.30	1.45
KC968	*csrD::kan, mreB'::msfGFP-mreB'-R124C*	105 min outgrowth in fresh LB	3, 5f	4.09	1.15

KC969	*csrD::kan, mreB'::msfGFP-mreB'-A174T*	120 min outgrowth in fresh LB	3, 5f	3.06	1.58
KC707	KC507 with *rodZ::kan* by P1 transduction from KEIO library strain JW2500	4 h outgrowth in fresh LB	5g	2.59	2.17
KC708	NO34 with *rodZ::kan* by P1 transduction from KEIO library strain JW2500	4 h outgrowth in fresh LB	5g	3.22	2.58
KC692	KC708 + plasmid P*mreB'::msfGFP-mreB'-CD*	90 min outgrowth in fresh LB	5a-b, g	3.83	2.81
KC694	KC708 + plasmid P*mreB'::msfGFP-mreB'-E276D-CD*	90 min outgrowth in fresh LB	5a-d, g	4.17	2.30
KC710	KC707 + plasmid P*mreB'::msfGFP-mreB'-E276D-CD*	90 min outgrowth in fresh LB	5c-d, g	1.87	1.41

Supplementary Table 3: Description of MD simulation systems from this study.

Name	Structure source	Ligand	Atoms (x1000)	Time (ns)	Source
2-MreB ATP	PDB: 1JCG	MreB: 2x ATP and 2x Mg^{2+} RodZ: none	95	140.8	[4]
2-MreB ADP	PDB: 1JCG	MreB: 2x ADP and 2x Mg^{2+} RodZ: none	95	68.0	[4]
2-MreB ATP + 2-RodZ, replicate 1	PDB: 2WUS (RodZ) and 1JCG (MreB)	MreB: 2x ATP and 2x Mg^{2+} RodZ: 2x	143	53.4	This work
2-MreB ATP + 2-RodZ, replicate 2	PDB: 2WUS (RodZ) and 1JCG (MreB)	MreB: 2x ATP and 2x Mg^{2+} RodZ: 2x	143	50.0	This work
2-MreB^{E266D} ATP, replicate 1	PDB: 1JCG	MreB: 2x ATP and 2x Mg^{2+} RodZ: none	107	60.1	This work
2-MreB^{E266D} ATP, replicate 2	PDB: 1JCG	MreB: 2x ATP and 2x Mg^{2+} RodZ: none	107	51.6	This work
2-MreB^{A162T} ATP, replicate 1	PDB: 1JCG	MreB: 2x ATP and 2x Mg^{2+} RodZ: none	107	63.1	This work
2-MreB^{A162T} ATP, replicate 2	PDB: 1JCG	MreB: 2x ATP and 2x Mg^{2+} RodZ: none	107	49.2	This work
2-MreB^{D72E} ATP, replicate 1	PDB: 1JCG	MreB: 2x ATP and 2x Mg^{2+} RodZ: none	107	66.5	This work
2-MreB^{D72E} ATP, replicate 2	PDB: 1JCG	MreB: 2x ATP and 2x Mg^{2+} RodZ: none	107	49.2	This work
2-MreB^{R112C} ATP, replicate 1	PDB: 1JCG	MreB: 2x ATP and 2x Mg^{2+} RodZ: none	107	66.5	This work
2-MreB^{R112C} ATP, replicate 2	PDB: 1JCG	MreB: 2x ATP and 2x Mg^{2+} RodZ: none	107	53.4	This work

Supplementary Figures

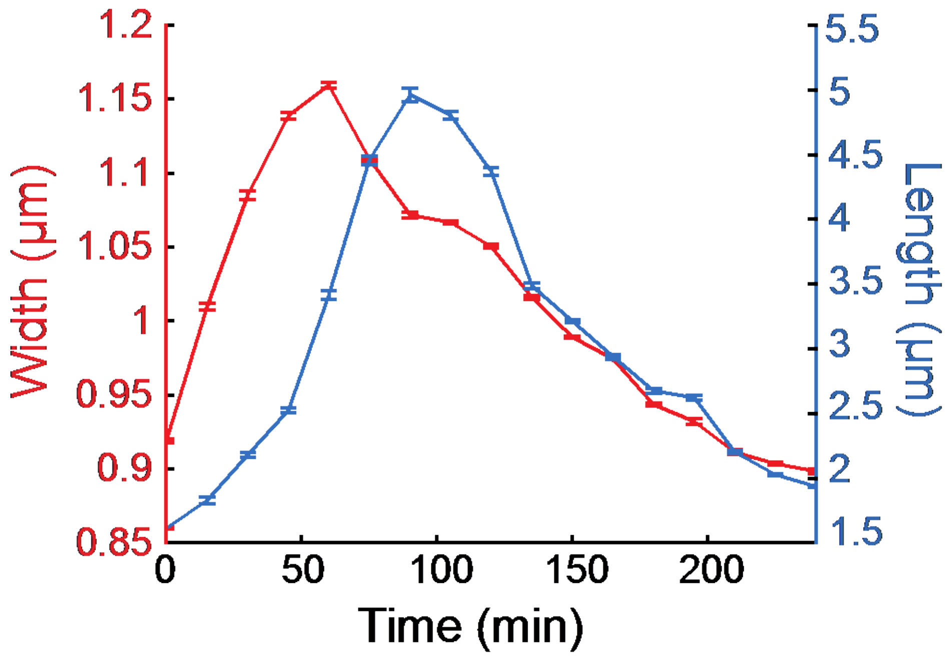

Supplementary Figure 1: Time course of cellular dimensions for wild-type *Escherichia coli* (unlabeled MreB).

To monitor cell shape as a function of cell density, we back-diluted an overnight culture of wild-type *E. coli* grown in lysogeny broth (LB) 1:200 into fresh LB in a test tube. Every 15 min, we extracted a small sample and imaged cells on an agarose pad with phase-contrast microscopy to measure cell shape (Methods). Error bars represent standard error with $n > 800$ cells.

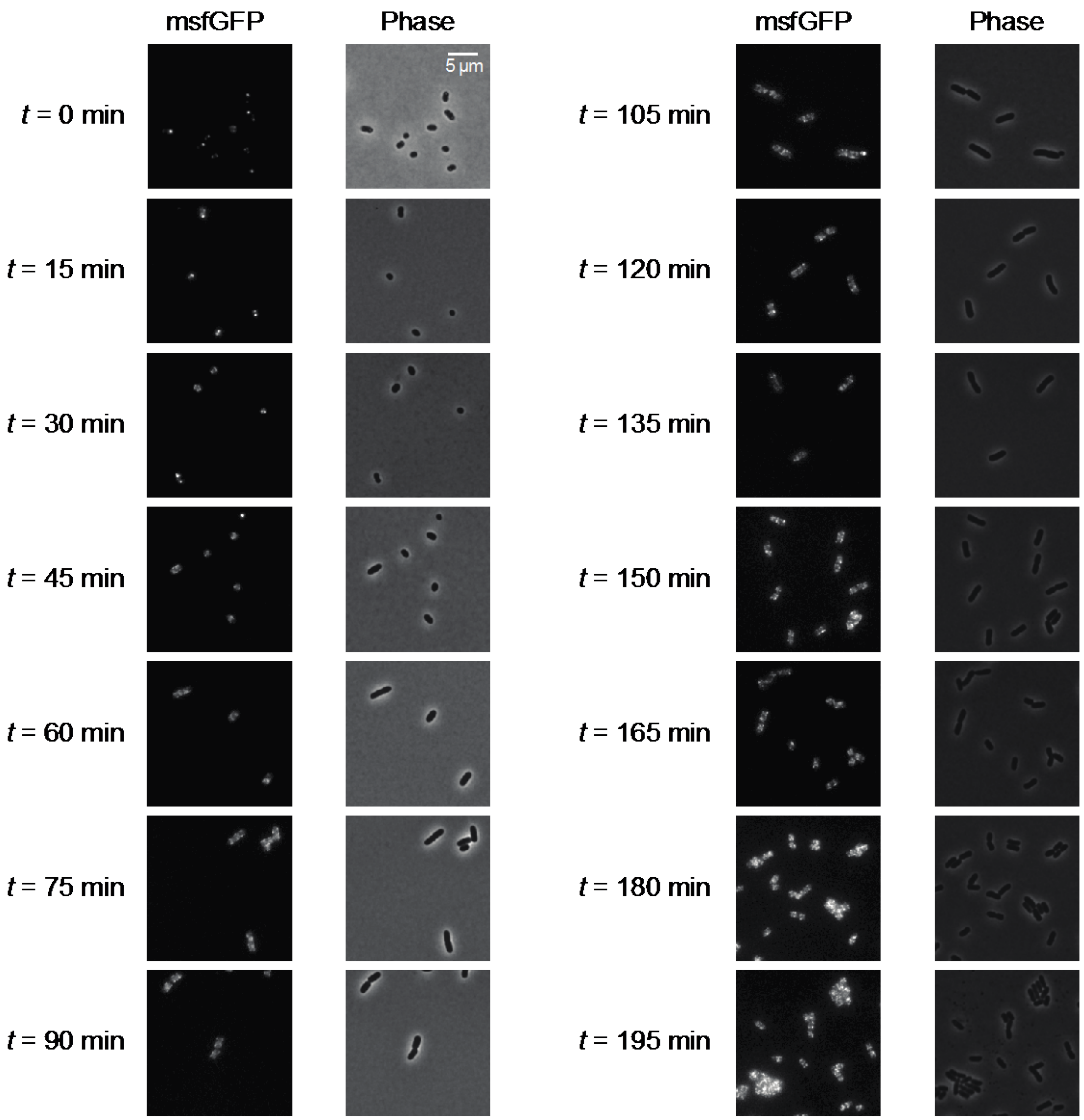

Supplementary Figure 2: Example of typical fields of view for time course in Figure 1.

To monitor cell shape as a function of growth phase during outgrowth from stationary phase, we back-diluted a 24-h culture of KC356 cells expressing an MreB-msfGFP sandwich fusion grown in LB 1:200 into fresh LB in a test tube. Every 15 min, we extracted a sample and imaged cells on an agarose pad. A representative phase-contrast and epifluorescence image from the same field of view from each time point is shown. Scale bar is 5 μm.

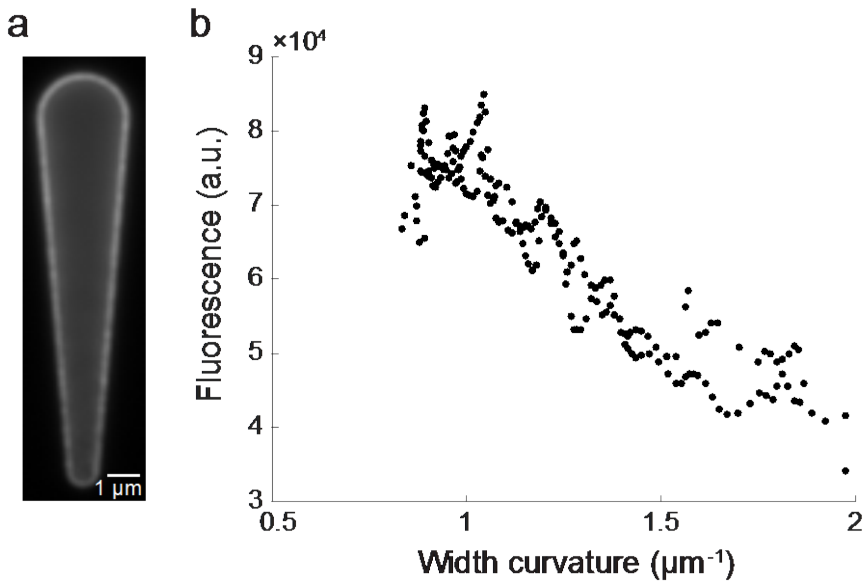

Supplementary Figure 3: Estimate of fluorescence enrichment expected due to optical artifacts in cells of varying width.

a) To test the effect of varying width in the context of constant contour curvature, a simulated epifluorescence image focused on the midplane of a cone with uniformly labeled fluorophores was generated using the software package *BlurLab*[5], which takes as input a set of locations of fluorescent molecules and convolves these locations with a point spread function to generate a simulated image. Scale bar is 1 µm.

b) A set of points along the contour of the simulated epifluorescence image was extracted using *Morphometrics* (Methods), and the relative fluorescence of each point on the contour was plotted against the width of the cone at that point. Artifacts relating solely to width are expected to contribute ~20% change in MreB enrichment for a cell with radial curvature of 1.2-2 μm^{-1}, which is less than the observed curvature enrichment of MreB for a constant contour curvature (Fig. 1f).

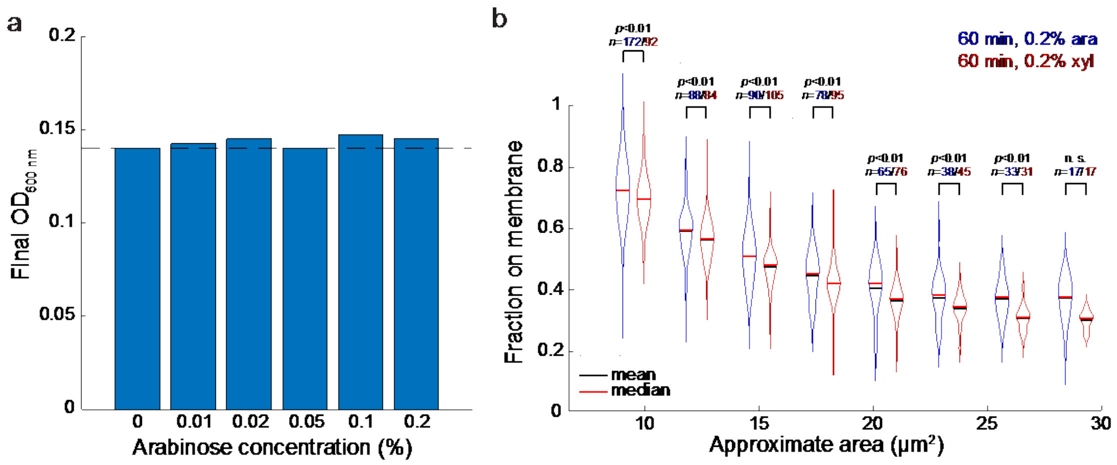

Supplementary Figure 4: Arabinose induction of RodZ alters MreB behavior in the absence of substantial cell growth.

(a) The optical density of all cultures from Fig. 2d following 60 min of exposure to varying concentrations of arabinose in spent media. Dashed line represents the OD of the culture prior to arabinose exposure.

(b) Cells with *rodZ* expression driven by P_{ara} were grown with 0.2% arabinose or 0.2% xylose. Both populations of cells were spheroidal. Cells of similar area were binned. For each bin, the fraction of MreB bound to the membrane was calculated as the sum of MreB on the periphery divided by the total fluorescence. The distribution of bound MreB was compared for each pair of bins across the two conditions. For every bin of cell areas, cells grown with arabinose exhibited a higher fraction of MreB bound to the membrane. Statistical significance was calculated with the one-tailed, two-sample Kolmogorov-Smirnov test.

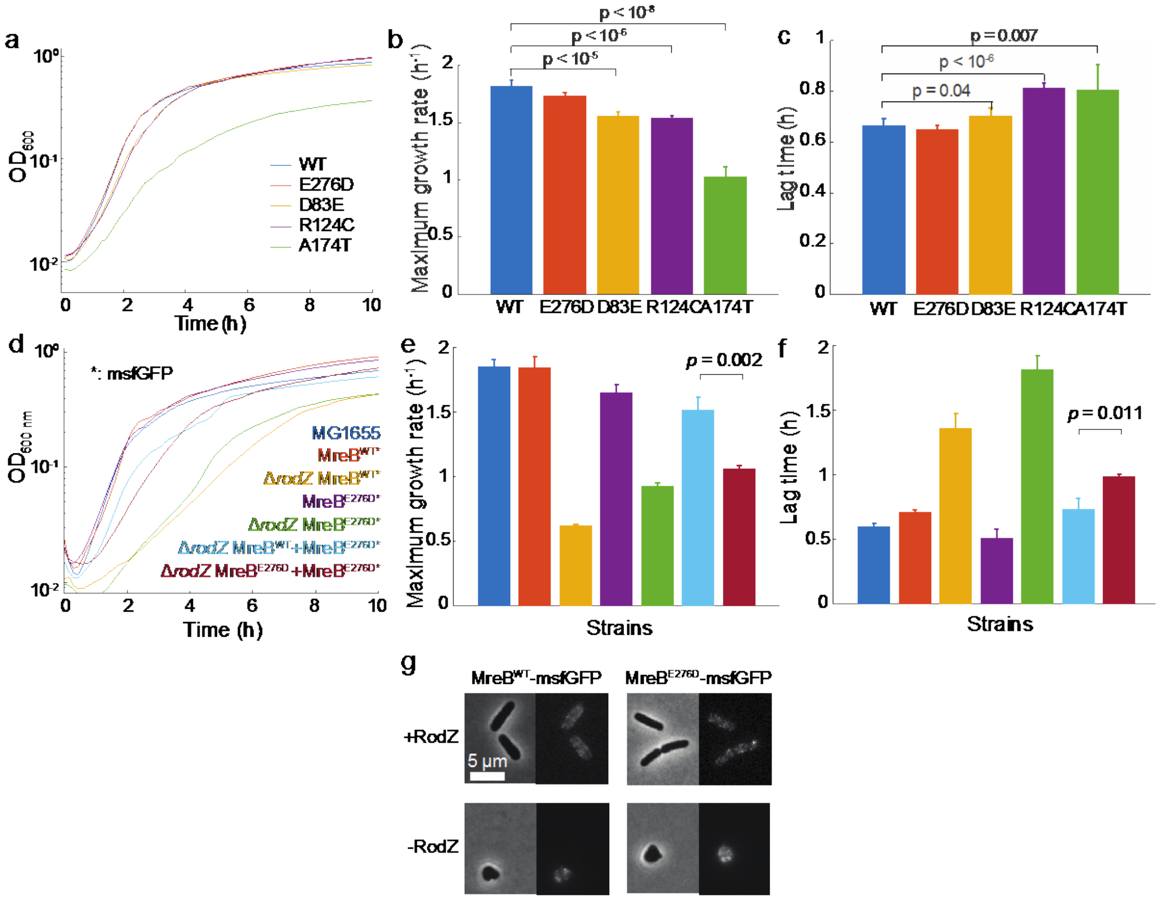

Supplementary Figure 5: Growth dynamics of strains.

a) To measure growth curves, strains were grown overnight in LB with appropriate antibiotics from frozen stocks. They were then diluted 1:200 into 200 µL of fresh LB with appropriate antibiotics in a transparent 96-well plate. Cultures were grown in a Tecan M200 Pro plate reader at 37 °C with continuous shaking and OD_{600} measured every 7.5 min. Each growth curve is the average of four replicates.

b) Maximum growth rates for the growth curves in (a) were determined by the maximum slope of ln(OD) versus time after two rounds of moving-average filtering with a sliding window of five time points. D83E, R124C and A174T have lower maximum growth rates compared to wild-type

strain (Student's *t*-test, $n = 4$). Data points for the bar graphs are represented by mean ± standard error of mean (s.e.m.) with $n = 4$.

c) The duration of lag phase for the growth curves in (a) was calculated as the time at which a line extrapolated from the point with maximum growth rate intersected with the initial OD, where initial OD was defined as the minimal OD across the curve, after subtracting the OD reading from a blank well. The mutant strains D83E, R124C and A174T have significantly longer lag time compared to the wild-type strain (Student's *t*-test, $n = 4$). Data points for the bar graphs represent by mean ± s.e.m. with $n = 4$.

d) Growth curves for Δ*rodZ* strains. Each growth curve is the average of four replicates. MG1655 is the unlabeled, wild-type strain. *: sfGFP fusion.

e) Maximum growth rates for the growth curves in (d). The Δ*rodZ* $MreB^{E276D}$+$MreB^{E276D*}$ strain grows significantly slower than the Δ*rodZ* $MreB^{WT}$+$MreB^{E276D*}$ strain (Student's *t*-test, $n = 4$). Data points for the bar graphs are represented by mean ± standard error of mean (s.e.m.) with $n = 4$.

f) The duration of lag phase for the growth curves in (d). The Δ*rodZ* $MreB^{E276D}$+$MreB^{E276D*}$ strain has significantly longer lag time compared to the Δ*rodZ* $MreB^{WT}$+$MreB^{E276D*}$ strain (Student's *t*-test, $n = 4$). Data points for the bar graphs represent by mean ± s.e.m. with $n = 4$.

g) Neither $MreB^{WT}$ nor $MreB^{E276D}$ could recover loss of rod shape due to deletion of *rodZ*. Scale bar is 5 µm.

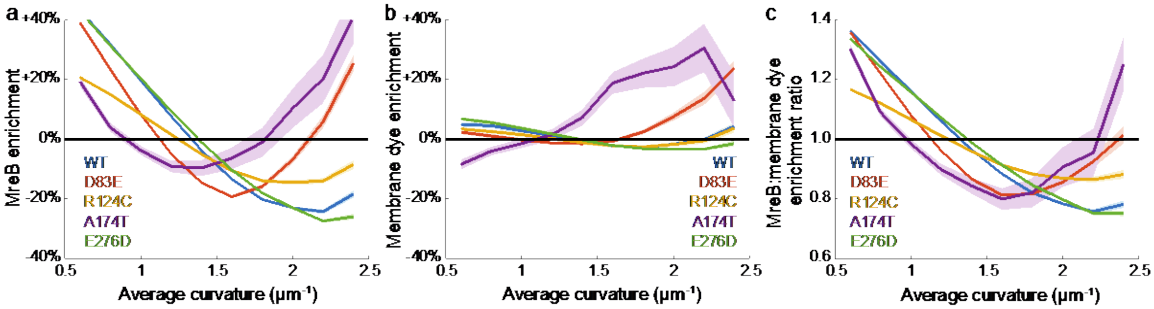

Supplementary Figure 6: Controlling for optical effects leads to qualitatively consistent measurements of MreB geometric sensing.

(a) MreB curvature enrichment of five *mreB* alleles reproduced from Fig. 3d. Shaded areas represent standard deviation of enrichment from resampled data for each strain.

(b) Curvature enrichment of fluorescence signal from the membrane dye FM4-64. Shaded areas represent standard deviation of enrichment from resampled data for each strain.

(c) Ratio of MreB fluorescence to membrane dye fluorescence from respective bins in (a) and (b) demonstrates that controlling for optical effects due to diverse cell morphologies leads to qualitatively similar MreB curvature enrichment profiles. The only major changes are at high average curvature for MreB^{D83E} and MreB^{A174T} cells. Shaded areas represent standard deviation of enrichment from resampled data for each strain.

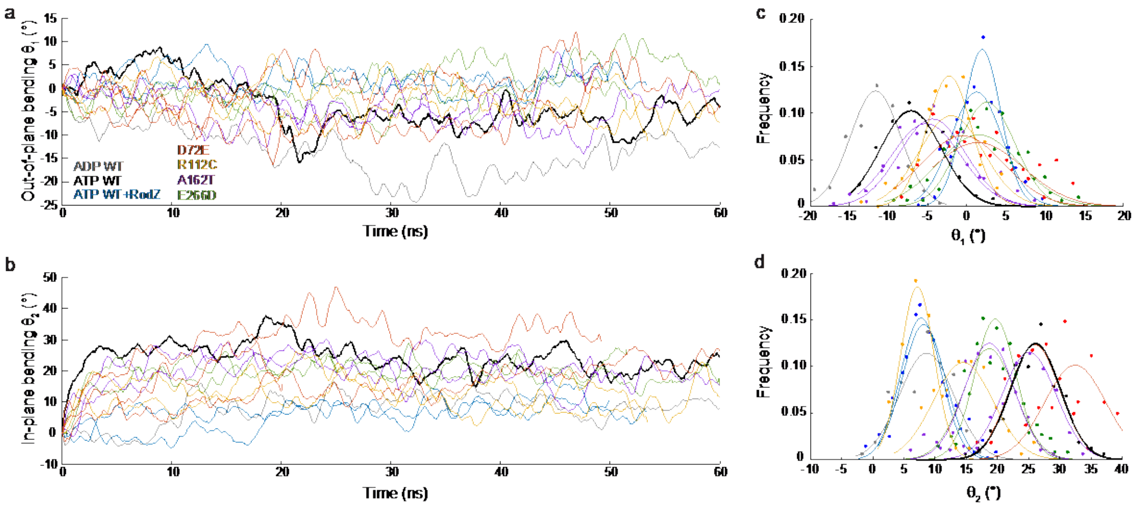

Supplementary Figure 7: Trajectories of MD simulations of MreB dimers.

a) The trajectories of bending angles between subunits of *Thermatoga maritima* MreB dimers across multiple conditions and replicates show that simulations reached a steady state within ~50 ns. When both ATP-bound MreB subunits were bound to RodZ, the dimer maintained a lower absolute value of θ_1 (out-of-plane of the membrane) than ATP-bound MreB subunits in the absence of RodZ (thick black lines). Similarly, all MreB point mutants investigated in our manuscript exhibited reduction in absolute θ_1 values relative to ATP-bound wild-type MreB in the absence of RodZ.

b) ATP-bound MreB dimers bound to two RodZ subunits maintained a lower absolute value of θ_2 (in-plane) than ATP-bound MreB dimers in the absence of RodZ. Similarly, nearly all replicates of MreB point mutants investigated in the manuscript exhibited reduction in absolute θ_2 values relative to ATP-bound wild-type MreB in the absence of RodZ.

c) Histograms of θ_1 (circles) from the last 30 ns of the trajectories in (a), and Gaussian fits to the histogram. A good fit indicates that the distribution of angles has reached equilibrium. The E266D (green, corresponding to $MreB^{E276D}$ in *E. coli*) had a quantitatively similar distribution to MreB-ATP bound to RodZ (blue).

d) Histograms of θ_2 (circles) from the last 30 ns of the trajectories in (b), and Gaussian fits to the histogram.

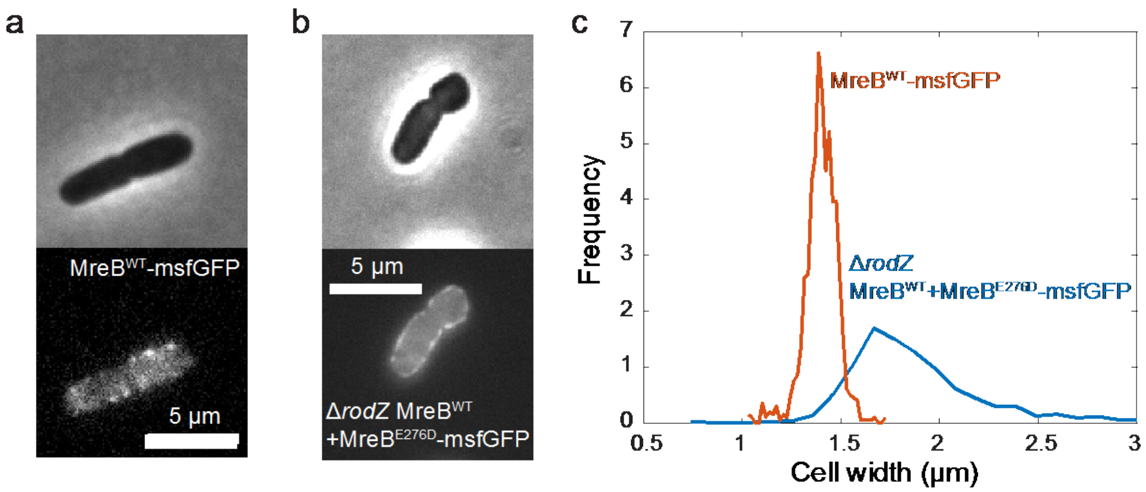

Supplementary Figure 8: Comparison of width of Δ*rodZ* MreB^{WT}+MreB^{E276D}-sfGFP with MreB^{WT}-msfGFP cells.

a) Phase and epifluorescence images of a typical *E. coli* cell with a single chromosomal copy of wild-type MreB fused to msfGFP. Scale bar is 5 µm.

b) Phase and epifluorescence images of a typical *E. coli* cell with a chromosomal copy of wild-type MreB as well as a plasmid-borne copy of MreB^{E276D}-msfGFP. Scale bar is 5 µm.

c) Distribution of cell widths of wild-type and Δ*rodZ* MreB^{WT}+MreB^{E276D*} cells in exponential phase shows that Δ*rodZ* MreB^{WT}+MreB^{E276D*} cells are wider on average and have a broader distribution of cell widths.

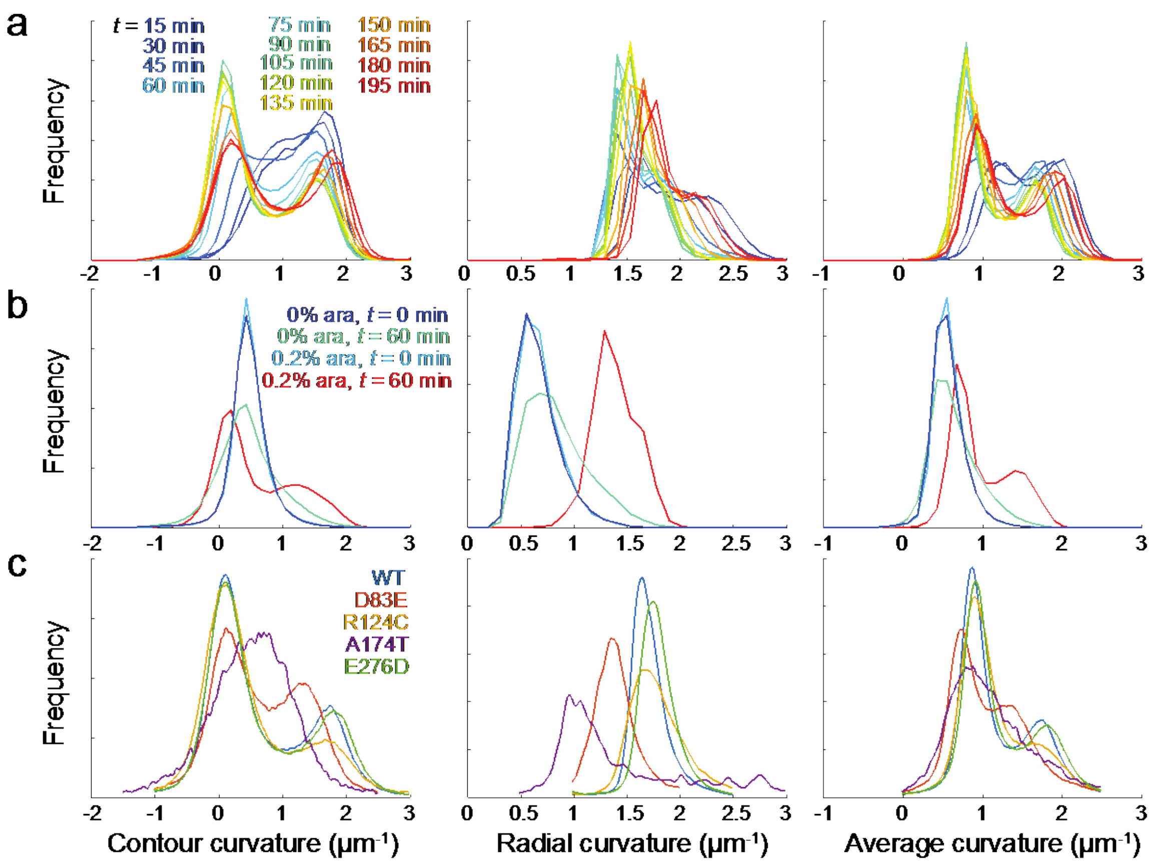

Supplementary Figure 9: Histograms of curvature values for populations of cells corresponding to the major figure panels in the main text. For the contour curvature (left), a peak at zero curvature represents straight regions of rod-shaped cells, while a second peak at positive curvature represents the poles. A single peak at positive curvature would represent more spheroidal cells. The spread of the radial curvature distribution (middle) represents the range of local cell widths. The average curvature values were estimated as the average of the contour and radial curvatures at each point along the contours.

a) Data appearing in Figure 1.

b) Data appearing in Figure 2.

c) Data appearing in Figure 3.

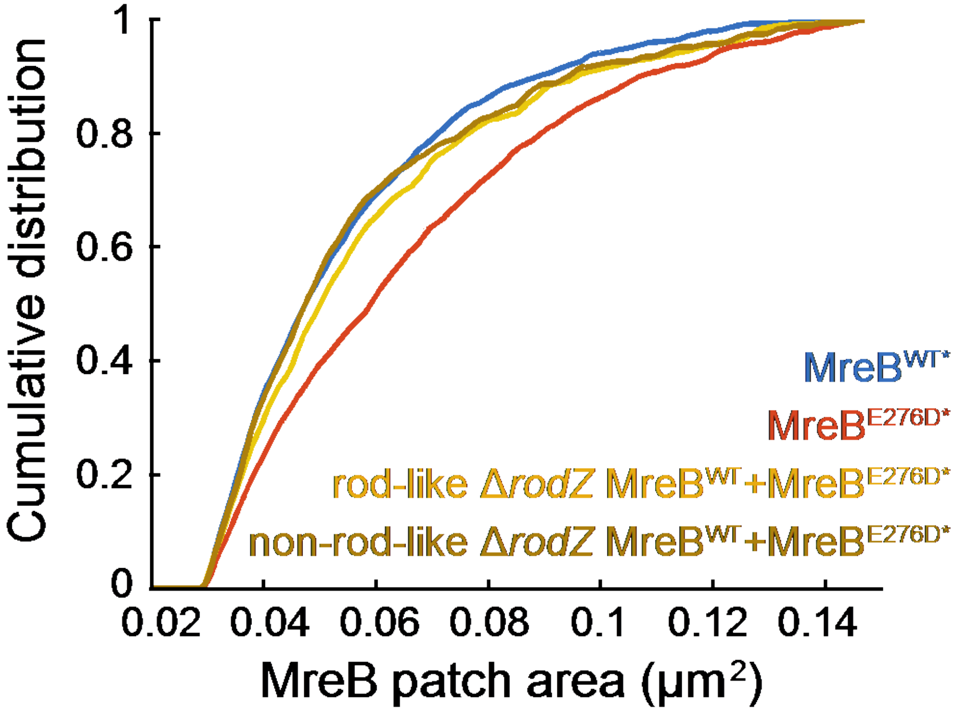

Supplementary Figure 10: Rod-like MreB^{WT}+MreB^{E276D}-msfGFP cells exhibit statistically significantly larger patch sizes than non-rod-like cells.

When divided into rod-like (yellow) and non-rod-like (brown) populations, the MreB^{WT}+MreB^{E276D}-msfGFP strains exhibited statistically significant differences in the distributions of MreB patch sizes; $p < 0.0001$ by the two-sample Kolmogorov-Smirnov test. *: msfGFP.

Supplementary References

1. Ouzounov, N. *et al.* MreB Orientation Correlates with Cell Diameter in Escherichia coli. *Biophysical Journal* **111**, 1035-1043 (2016).
2. Baba, T. *et al.* Construction of Escherichia coli K-12 in-frame, single-gene knockout mutants: the Keio collection. *Molecular systems biology* **2**, 2006 0008 (2006).
3. Bendezú, F. O., Hale, C. a., Bernhardt, T. G. & de Boer, P. a. J. RodZ (YfgA) is required for proper assembly of the MreB actin cytoskeleton and cell shape in E. coli. *The EMBO journal* **28**, 193-204 (2009).
4. Colavin, A., Hsin, J. & Huang, K. C. Effects of polymerization and nucleotide identity on the conformational dynamics of the bacterial actin homolog MreB. *Proceedings of the National Academy of Sciences of the United States of America* **111**, 3585-3590 (2014).
5. Ursell, T. S. *et al.* Rod-like bacterial shape is maintained by feedback between cell curvature and cytoskeletal localization. *Proceedings of the National Academy of Sciences of the United States of America* (2014).
